# Antecedents of attitude and their impact on behavioral intention in the staycation context

**DOI:** 10.3389/fpsyg.2022.996788

**Published:** 2022-09-06

**Authors:** Yating Zhang, Huawen Shen, Jiajia Xu, Stella Fang Qian

**Affiliations:** ^1^Faculty of International Tourism and Management, City University of Macau, Macau, Macau SAR, China; ^2^Faculty of Business Administration, University of Macau, Macau, Macau SAR, China

**Keywords:** staycation, mindsponge mechanism, reduced risk perception, benign envy, perceived benefits, attitude, behavioral intention

## Abstract

The year 2020 and 2021 have been decimated by the pandemic, leading to outbound vacations largely scrapped. Staycation, a typical domestic journal, has then been adopted by those who are tired of self-isolation for so long. This study aims to explore and assess the drivers exerting impact on attitude of tourists toward staycation and the interrelationship among the research constructs is also examined. A quantitative analysis is employed for evaluating the roles of reduced risk perception, benign envy, and perceived benefits as they exert the effect on attitude toward staycation. An online questionnaire survey was used, and a total of 213 samples were collected from target respondents in Hong Kong, which were still under lockdown at the time of the study. The results of the study showed that reduced risk perception, benign envy as well as perceived benefits will influence tourists’ attitude toward staycation. The managerial and theoretical implications of the results are discussed based on the significant relationships identified in the study.

## Introduction

Since the beginning of the 20th century, there have been two world wars, three serious economic crises, and four pandemics. Each time, tourism has been one of the hardest hit industries. Fortunately, the tourism industry has taken various measures to overcome the challenges. For example, during the financial crisis from 2007 to 2008, with the low growth of inbound tourism, the relevant authorities (Sheldon and Dwyer, 2010) implemented various measures, such as focusing on national tourism, to cope with this challenge. At the time, staycations, a type of domestic tourism, were popular in the United States and the United Kingdom. A staycation as a short period of time, sometimes a vacation, for being homebound, it can be seen as proximity tourism (Wixon, 2009; Jeuring and Haartsen, 2017). Staycation also refers to a vacation taken at or near one’s home (De Bloom et al., 2017), while considering “staying voluntarily,” another name for staycation, as a local tourism market share. Today, the world is still grappling with the ravages of the deadly COVID-19 pandemic, and staycations have become popular again. For instance, despite their lockdowns being lifted, some countries or regions, such as Iran, the United States, and Hong Kong, faced a second wave of COVID-19 infection (Huang et al., 2020). Therefore, to boost the hospitality industry, staycation has become a major stimulus in a cratering tourism economy (Grech et al., 2020). One of the main advantages of a staycation is to save money. Compared with a long-haul holiday, a staycation significantly reduces accommodation and meal costs, as well as travel expenses. With the global economy back in the doldrums, along with the negative impacts of COVID-19, it is not difficult to understand the renewed interest in staycations. The growing number of staycation booking websites (STAYCATION Inc., 2020) and the number of people vacationing locally reflect the popularity of staycations. In addition, governments encourage their citizens to choose staycations to improve the local economy. For example, the Maltese government offers five €20 vouchers to all residents over the age of 16 to spend on staycations (Grech et al., 2020). Similarly, Belgium offers 10 free train tickets to every citizen for staycations to improve the domestic economy (Broom, 2020).

However, to the best of our knowledge, no study has examined factors which will influence the attitude of tourists toward staycation. Taking cognizance of the current paucity of in this area, this study proposes to identify the drivers exerting impact on attitude of tourists toward staycation and the interrelationship among reduced risk perception, benign envy, perceived benefits as well as tourists’ behavioral intention will also be investigated. The result of the study will enrich theoretical understanding of the examined variables in the context of staycation where there is still lack of dedicated research. Moreover, managerial implication for the development of this mode of travel can be derived.

## Literature review

### Staycation

Wixon (2009) defined a staycation as “a short period of time, sometimes a vacation, for being homebound, or staying in neighborhood by establishing the atmosphere of a conventional holiday-making.” Vačková (2009) offered a comparable definition, expressing that a staycation is a movement of staying at home instead of making a trip to another objective and exploring the neighborhood environment.

During a staycation, travelers experience different assortments of changes to adapt themselves to a holiday-alike environment awash with leisure time and rich variety of activities (Sharma, 2009). De Bloom et al. (2017) asserted that staycation encompassed the variety of leisure-embedded activities close to the domicile required limited volume of traveling. The fundamental vicissitude of the very existence of tourism and hospitality brought about by COVID-19 since early 2020 has further practically positioned the concept of staycation as the antithetical to overseas and long-haul travel, concerning holiday trips within the destination or the country at large (Yan et al., 2022). Therefore, a staycation can be seen as proximity tourism (Jeuring and Haartsen, 2017), which is akin to a day trip during which travelers can return home or spend the night in a hotel. Staycations are already popular around the world, despite the perceived risk, uncertainty, and insecurity of the COVID-19 pandemic that still prevail. In mid-2020, the Hong Kong Hotels and Catering Practitioners Association (Hotel Industry Association) stated that because the global epidemic was not fully contained and the tourism industry had yet to recover, Hong Kong hotels would launch staycations, long-term rentals, and shared offices to expand their businesses (Moon and Chan, 2022).

### Mindsponge mechanism

Vuong and Napier (2015) proposed the mindsponge mechanism for explaining how and why an individual “learns and unlearns” culture values, which helps better understand the complexity of acculturation in a global context. A person’s attitudes or behavioral intentions are the products of many mental processes involving various types of information, thoughts, and feelings. According to articulation of the mindsponge mechanism, information is absorbed into or ejected out of a person’s mindset, which are a set of core values or beliefs that influence the subsequent thinking and behaviors of the person because of the multi-filtering system (e.g., subjective cost-benefit judgments) of the person toward the given information (Vuong and Napier, 2015). Safety is one of the basic needs of humans due to the survival instinct, while entertainment is another important need to improve humans’ well-being (Vuong, 2022). Such needs create the demand for information that help satisfy the demand for safety and well- being, making people perceive information related to staycation as beneficial or hold more favorable attitude toward staycation and absorb them easily into their core values (or become the person’s behavioral intention). Thus, The mindsponge mechanism boasts its merits in approximating, evaluating and estimating the perceptions and behavioral intentions of tourists, with the integration of social and psychological perspectives (Nguyen et al., 2021). It is deemed by this study to serve as tenable platform upon which the interrelationships of the research variables can be constructed.

### Reduced risk perception

The concept of “risk perception” was first introduced by Bauer (1960) to analyze consumer behavior. According to some studies, perceived risk, a significant factor influencing people’s behavior (Weinstein, 1988), refers to people’s subjective assessment of risk in a threatening situation (Ariffin et al., 2018; Mohseni et al., 2018). In the context of the COVID-19 pandemic, perceived risk is represented by individual susceptibility to infection and its perceived severity (Kim and Kim, 2018; Yıldırım and Güler, 2020). The former can be defined as the perceived risk of contracting a disease while the latter refers to personal perception of the seriousness about a disease (Bruine de Bruin, 2020), which is the definition adopted in the context of the COVID-19 pandemic in this study. Perceived seriousness and perceived susceptibility can also be categorized as cognitive representations of risk perception, one of the dimensions of risk perception examined by researchers (Brug et al., 2004). Another dimension examined in the literature is affective risk perceptions, referring to an individualusness and perceived susceptibility can also be ease Sjöberg, 1998).

In the context of tourism, risk has been testified as a primary concern for international tourists (Ugur and Akbiyik, 2020). A decline in travel demand is a consequence of the perceived risk of travel, such as terrorism (Karl and Schmude, 2017), disease (Pine and McKercher, 2004; Leggat et al., 2010; Yanni et al., 2010; Chua et al., 2020), natural disasters (Park and Reisinger, 2010; Karl and Schmude, 2017), and mega events (Schroeder et al., 2013). As people inherently seek to meet their safety needs, they can be strongly influence by safety and security issues when making travel decisions under risk uncertainty. Furthermore, the experiential and intangible traits of tourism invariably contribute to tourists’ perception of higher levels of unsystematic risk. Therefore, positive feelings and travel intention can be generated when people’s level of risk perception is low (Lepp et al., 2011).

### Benign envy

Previous research has shown that friends or even strangers who publish tourist photos on WeChat or other social media apps such as TikTok and Facebook can lead to subsequent social comparison among users (Pera, 2018; Wong et al., 2019; Pang, 2020; Anderson, 2021). In accordance to social comparison theory (Festinger, 1954), social comparison occurs when people see travel-related information posted by friends on social networking sites, resulting in an overwhelming desire to become a follower; that is, social comparison can trigger BE. BE is a type of feeling in which a person is seen by others as having good results, accomplishments, or possessions (Khan et al., 2017).

### Perceived benefits

Benefits refer to the rewards obtained by a consumer after using a product or experiencing a service (Gutman, 1982). In the tourism context, based on the literature (Westman et al., 2008; Yolal et al., 2009; Han et al., 2014; Carlson, 2015; Chen and Petrick, 2016), the PB of a staycation are socialization, escape and excitement, and family togetherness.

Leveraging a series of studies, Westman and her coworkers investigated the effect of vacations on job burnout. Their results showed that vacations reduced job stress and job burnout in 21 respondents (Westman and Eden, 1997; Westman and Etzion, 2001, 2002; Westman et al., 2008). Similarly, Fritz and Sonnentag (2006) and Sonnentag and Fritz (2007) illustrated that recovery from experiences in vacation (such as psychological disengagement at work, relaxation experience, and perceived control during vacation) could promote employees’ physical and mental health by offering the internal and external resources.

### Attitude toward staycations

An attitude can be simply defined as a mental propensity to behave in a certain way due to experience and behavior. Eagly and Chaiken (1993) defined an attitude as an individualas tendency to rate a particular entity favorably or unfavorably. The more comprehensive definition proposed by Kurniawati et al. (2017) states that attitudes consist of affective, cognitive, and behavioral components, which reflect an individual’s beliefs about the targeted object. Attitude is a defining part of understanding the motivation behind tourist behavior (Gruen et al., 2005). Accordingly, an attitude toward a certain way of travel is an important premise for the following behavior in the context of tourism.

### Behavioral intention

Behavioral intention (BI) is an indication that a person is ready to perform a given action (Ajzen, 2002). In the context of travel, BI is defined as a person’s desire or intention to travel. In addition, according to the study conducted by Baker and Crompton (2000), BI refers to whether a tourist will visit a given destination and their intention to return. Accordingly, BI in this study refers to the desire and willingness to have a staycation.

## Research model and hypotheses

**FIGURE 1:**
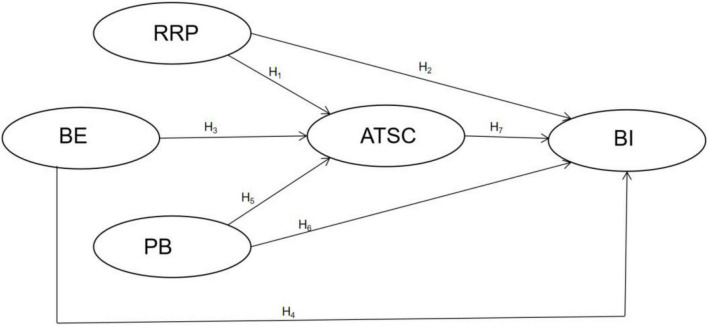
The research model. RRP, reduced risk perception; BE, benign envy; PB, perceived benefits; ATSC, attitude toward staycations; BI, behavioral attitude.

The model that integrates the seven hypotheses tested in this study regarding the roles of RRP, BE, PB, ASTC, and BI is shown in Figure 1.

### Effect of reduced risk perception on attitude toward staycations and behavioral attitude

In most studies on consumer behavior, attitudes are invariably negatively affected by perceived risk (Habibi and Rasoolimanesh, 2020). In the context of this study, those who feel at risk of infection will certainly take measures not to travel to avoid getting infected (Gostic et al., 2020; Neuburger and Egger, 2020). Therefore, I propose that RRP is related to peopleks attitudes and BI. Accordingly, in this study, the following hypotheses are proposed to test the effect of RRP on ATSC and BI.

*H1: RRP has a positive impact on ATSC.*

*H2: RRP has a positive impact on BI.*

### Effect of benign envy on attitude toward staycations and behavioral attitude

Based on social comparison theory, BE can be triggered by upward interpersonal comparison (Van de Ven, 2017; Meier and Schafer, 2018; Latif et al., 2020; Van de Ven and Zeelenberg, 2020). Accordingly, viewing social comparison information such as tourist selfies on social media is expected to trigger people’s BE, which in turn will affect their BI (Van de Ven and Zeelenberg, 2020). However, as no previous study has confirmed the positive relationship between BE and attitudes, this study tested this relationship based on the following hypotheses.

*H3: BE has a positive impact on ATSC.*

*H4: BE has a positive impact on BI.*

### Effect of perceived benefits on attitude toward staycations and behavioral attitude

A large number of studies in various contexts have confirmed the positive relationship between specific benefits, attitude, and BI. For example, Patro (2019), in his study of online shopping, found that cash saving was a critical antecedent of attitude. Habibi et al. (2018) found a positive relationship between perceived medical quality, the most important measure of PB, and attitude toward medical tourism. In the context of a cruise service, Lemmetyinen et al. (2016) illustrated that the benefits of relaxation had a positive impact on attitude and BI. Accordingly, I propose the following hypotheses for the relationship between PB, TSC, and BI:

*H5: PB has a positive impact on ATSC.*

*H6: PB has a positive impact on BI.*

### Attitude toward behavioral attitude

In the context of tourism, attitude is a key variable that influences tourists’ future BI (Ponte et al., 2015; Jeng and Lo, 2019). Many studies have established that purchase intention is positively germane to attitude (Gan and Wang, 2017; Salehzadeh and Pool, 2017; Chae et al., 2020). Therefore, the final hypothesis tested in this study is as follows:

*H7: ATSC has a positive impact on BI.*

## Methodology

Based on the model presented in Figure 1, this study sought to answer the following two questions: (1) What are the impacts of the three antecedents (RRP, BE, and BI) of attitude on ATSC and BI. (2) What is the relationship between ATSC and BI? To address these questions, a questionnaire designed was containing five sets of questions (22 items) based on the model’s five constructs. A 7-point Likert-type scale was used to answer the questions, ranging from 1 (strongly disagree) to 7 (strongly agree). The respondents were required to complete the questionnaire at the basis of their perceptions of and experience with staycations.

### The survey instrument

To develop the questionnaire, this manuscript adopted the items from the scales used by previous researchers and modified them to fit the staycation context (as shown in Table 1). The eight items used to measure perceived risk in the study by Bae and Chang (2020) were used to measure RRP in this study (i.e., “there is a low likelihood of acquiring COVID-19 in general”; “there is a low likelihood that I will acquire COVID-19 compared to other people”; “there is a high likelihood of acquiring COVID-19 compared to other diseases”; “there is a low likelihood of dying from COVID-19”; “I am not worried that I will contract COVID-19; I am not worried about my family members contracting COVID-19”; “I am not worried about COVID-19 occurring in my region”; “I am not worried about COVID-19 emerging as a health issue”). To measure BE, four items (i.e., “I am motivated to work hard”; “I compliment my friend,” “I wish it were me,” and “I want to follow my friend’s travel posts”) were used based on the study by Jing et al. (1996). To measure PB, three items related to socialization (Stollery and Jun, 2017), four items related to escape and excitement, and one item related to family togetherness were used (i.e., “for a chance to be with people who are enjoying themselves”; “to be with a people of similar interest”; “to be with people who enjoy the same things I do”; “for a change of pace from my everyday life”; “to have a change from my daily routine”; “to experience new and different things because I was curious”; “to get away from the demands of life because it is stimulating and exciting”; “because I thought the entire family would enjoy it, the family could do something together”) (Frochot and Morrison, 2000). ATSC was measured using a 4-item scale (i.e., “For me, a staycation would be extremely enjoyable”; “For me, a staycation would be extremely fun”; “For me, a staycation would be extremely pleasant”; “For me, a staycation would be extremely positive”) adapted from Hasan et al. (2019). The final variable, BI, was measured using three items (i.e., “I intend to have a staycation in the future”; “I predict that I would have staycation in the future”; “I am willing to have staycation in the future”) adapted from Ahn and Kwon (2020).

**TABLE 1:** Measurement items.

Latent variable	Measurement item	Source
**RRP**	RRP 1	Bae and Chang (2020)
	RRP 2	
	RRP 3	
	RRP 4	
	RRP 5	
	RRP 6	
	RRP 7	
	RRP 8	
**BE**	BE1	Jing et al. (1996)
	BE2	
	BE3	
	BE4	
**PB**	PB 1	Stollery and Jun (2017)
	PB 2	
	PB 3	
	PB 4	
	PB 5	
	PB 6	
	PB 7	
	PB 8	
**ATSC**	ATSC 1	Stollery and Jun (2017)
	ATSC 2	Frochot and Morrison (2000)
	ATSC 3	
	ATSC 4	Chen (2012)
**BI**	BI 1	(Chaulagain et al., 2019; Ahn and Kwon, 2020)
	BI 2	
	BI 3	

RRP, reduced risk perception; BE, benign envy; PB, perceived benefits; ATSC, attitude toward staycations; BI, behavioral attitude.

To develop the questionnaire, this manuscript referred to expert opinions to select appropriate proxy variables. To confirm the effectiveness of the measurement method, nine experts were invited from the tourism industry to complete the questionnaire. The nine experts were selected based on their work experience and related expertise in tourism management.

### Date collection and data analysis

To achieve the objectives of the study, this manuscript collected data using an online survey in Wenjuanxing^1^, an online crowd-sourcing platform in China. Surveys are typically used in social science research (Boonsiritomachai and Phonthanukitithaworn, 2019). The target respondents in this study were mainly from Hong Kong, which was still under lockdown at the time of the study. Over a period of 1 week (from 19th to 26th in December of 2021), 263 responses were received. In evaluating the questionnaires received, 50 questionnaires were found to be improper for further data analysis due to incomplete or incorrect information and were removed from the study, resulting in 213 samples. Data were analyzed following the principles and procedures of structural equation modeling (SEM). According to Kaplan (2000), SEM can perhaps be best defined as a class of methodologies that seeks to represent hypothesis about the means, variances, and covariance of observed data in terms of a smaller number of structural parameters defined by a hypothesized underlying model. The essence of applying SEM is handling structural relationship, especially relationships between latent constructs or variables (Nachtigall et al., 2003).

## Results

### Respondent demographics

As shown in Table 2, the sample was evenly split between men and women. The sample size was *N* = 213 (51.64% male, 48.36% female). There were two large age groups: the 26a30 age group, representing 35.68% of the sample, and the 31–40 age group, accounting for 29.11% of the sample. Among the respondents, 97.18% were full-time employed. Finally, the number of respondents with an undergraduate degree represented 67.14% of the sample.

**TABLE 2:** Demographic characteristics of the sample.

Variable	*N*	Percentage (%)
**Gender**		
Male	110	51.64
Female	103	48.36
**Age**		
Below 18	1	0.47
18–25	2	0.94
26–30	76	35.68
31–40	62	29.11
41–50	59	27.7
51–60	13	6.1
61 and above	0	0.00
**Education level**		
Senior Middle School Diploma	16	7.51
Junior or Vocational College	33	15.49
Bachelor’s degree/Undergraduate	143	67.14
Master’s degree and above	21	9.86
**Career**		
Full-time students	4	1.88
Employed	207	97.18
Houseperson	2	0.94
Retired	0	0.00
Other	0	0.00
**Income (RMB)**		
Below 3,500	18	8.45
3,501–4,999	80	37.56
5,000–7,999	85	39.91
8,000 and above	30	14.08

### Reliability analysis

The reliability of a scale is typically measured using Cronbach’s alpha. Cronbach’s alpha (reliability coefficient) values range between 0 and 1, indicating a reliable scale with satisfactory convergent validity (Hair et al., 1998). According to Hair et al. (1998), Cronbach.s alpha greater or equal to 0.80 indicates a reasonable scale, a value of 0.70 indicates an acceptable scale, and a scale with Cronbach’s alpha of 0.60 is defined as weak for exploratory purposes. Composite reliability (CR) is a preferred alternative to Cronbachus alpha for testing convergent validity in a reflective model because Cronbach’s alpha may overestimate or underestimate the reliability of a scale (Gliem and Gliem, 2003). CR ranges from 0 to 1, with 1 indicating perfect convergent validity. In a model suitable for exploratory purposes, CR should be equal to or greater than 0.6 (Chin, 1998; Hock et al., 2010). In this study, the internal consistency of the questionnaire was tested by examining the reliability of each part of the scale separately, and the test results are reported in Table 3; Cronbach s alpha for the scale was greater than 0.7, indicating high internal consistency.

**TABLE 3:** Reliability analysis results for each study variable.

Variable	CITC	Cronbach’s alpha when the item was deleted	Cronbach’s alpha
RRP1	0.75	0.90	0.91
RRP2	0.68	0.90	
RRP3	0.72	0.90	
RRP4	0.70	0.90	
RRP5	0.69	0.90	
RRP6	0.70	0.90	
RRP7	0.70	0.90	
RRP8	0.79	0.89	
BE1	0.78	0.82	0.87
BE2	0.73	0.84	
BE3	0.74	0.84	
BE4	0.69	0.86	
PB1	0.77	0.90	0.91
PB2	0.72	0.91	
PB3	0.70	0.91	
PB4	0.68	0.91	
PB5	0.74	0.90	
PB6	0.71	0.91	
PB7	0.70	0.91	
PB8	0.79	0.90	
ATSC1	0.73	0.84	0.87
ATSC2	0.71	0.85	
ATSC3	0.76	0.83	
ATSC4	0.73	0.84	
BI1	0.78	0.79	0.87
BI2	0.71	0.85	
BI3	0.77	0.80	

RRP, reduced risk perception; BE, benign envy; PB, perceived benefits; ATSC, attitude toward staycations; BI, behavioral attitude; CITC, correlation of item totals.

As shown in Table 3, Cronbachns alpha was 0.91 for RRP, 0.87 for BE, 0.91 for PB, 0.87 for ATSC, and 0.87 for BI; therefore, all Cronbach’s alpha values were greater than 0.7 and most were above 0.8, indicating that the latent variables in the questionnaire were well defined and the questionnaire was valid. As a result, the questionnaire used in this study had good reliability. Moreover, most of the Correlation of item totals (CITC) between observed variables and their latent variables are between 0.6 and 0.8. According to the requirement of greater than 0.5, the correlation coefficient of each observation variable and its latent variable is CITC more than 0.5, and most of them are between 0.6 and 0.8, which indicates that the latent variables of each question are well set and the reliability of the questionnaire is valid. At the same time, by excluding the observation variables, the concrete method is to delete each variable once. If the reliability index does not change after deletion, it is considered that the measurement question of the variable has good credibility.

### Validity analysis

#### Exploratory factor analysis

This study used SPSS Amos 21.0 to perform exploratory factor analysis (EFA) and confirmatory factor analysis (CFA). EFA is typically used to measure the structural utility of a scale and determine whether the measurement items for each latent variable are consistent with a stable structure; it is the most commonly used index to evaluate the efficiency of a scale. When using factor analysis for validity analysis, it is important to first determine whether the main conditions for factor analysis are met. One condition is that the Kaiser–Meyer–Olkin (KMO) value is greater than 0.7; the other is that the significance of Bartlett’s test of sphericity is less than 0.05. Satisfying these two conditions indicates that there is a strong correlation between the observed variables, which are suitable for factor analysis.

Table 4 presents the EFA results. Specifically, the KMO value was 0.938, which is significantly greater than the standard value of 0.70, and Bartlett’s test of sphericity was 3,961.381, with a *p* value smaller than 0.001, which was suitable for factor analysis. The principal component analysis method was adopted to extract factors with values greater than 1. As shown in Table 4, five common factors (RRP, BE, PB, ATSC, and BI) were extracted, and the cumulative sum of the squared rotated loadings was 68.961%, which is greater than 60%. Specifically, after rotation using the orthogonal rotation method, the 27 questionnaire items were classified into five factors. The factor loading of each item was greater than 0.5, indicating that the information contained in these five factors was comprehensive and there were no double factor loadings. In the case of high factor loadings, the observed variables were aggregated in each dimension according to the theoretical settings. Based on the above analysis, the questionnaire used in this study had good construct validity.

**TABLE 4:** Validity analysis results for each study variable.

	Component				
	
	1	2	3	4	5
RRP1	0.703				
RRP2	0.683				
RRP3	0.712				
RRP4	0.706				
RRP5	0.683				
RRP6	0.674				
RRP7	0.703				
RRP8	0.780				
BE1				0.783	
BE2				0.792	
BE3				0.765	
BE4				0.722	
PB1		0.622			
PB2		0.674			
PB3		0.558			
PB4		0.707			
PB5		0.593			
PB6		0.616			
PB7		0.550			
PB8		0.753			
ATSC1			0.736		
ATSC2			0.735		
ATSC3			0.800		
ATSC4			0.750		
BI1					0.838
BI2					0.827
BI3					0.840
Total	12.593	1.882	1.62	1.452	1.072
Cumulative%	19.208	35.297	47.665	59.432	68.961
KMO test	0.938				
Bartlett’s test	3,961.381 (*p* = 0.000)				

Extraction method: principal component analysis. Rotation method: varimax with Kaiser normalization. RRP, reduced risk perception; BE, benign envy; PB, perceived benefits; ATSC, attitude toward staycations; BI, behavioral attitude; KMO, Kaiser–Meyer–Olkin test.

#### Confirmatory factor analysis

**FIGURE 2:**
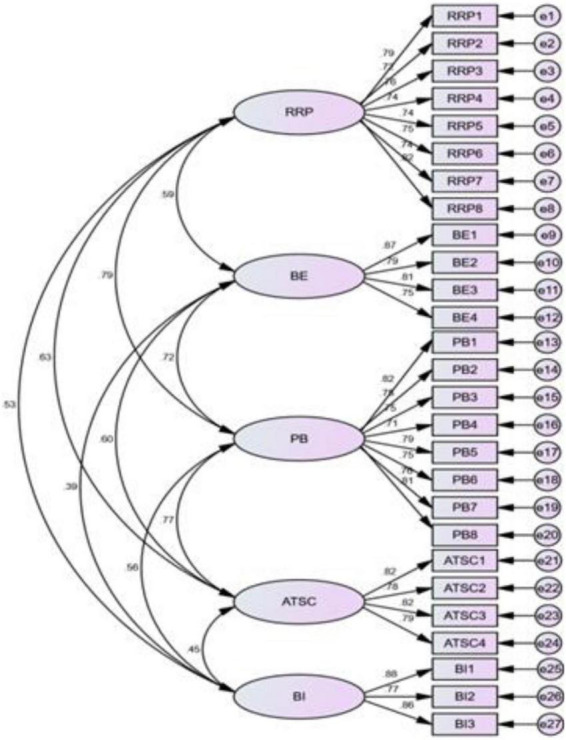
Representation of the CFA Model. RRP, reduced risk perception; BE, benign envy; PB, perceived benefits; ATSC, attitude toward staycations; BI, behavioral attitude.

This manuscript also used SPSS Amos 21.0 to perform CFA on the questionnaire used in this study, develop a confirmatory factor model based on the EFA results (shown on Figure 2), and determine whether the proposed model was appropriate by analysing the fit indices of the structural equation model. The fit indices reported in Table 5 show that the proposed model effectively measured the related latent variables.

**TABLE 5:** Model fit indices.

Reference indicator	χ^2^/*df*	GFI	AGFI	NFI	TLI	CFI	RMSEA
Statistics	1.711	0.836	0.803	0.871	0.934	0.941	0.058
Reference	<3	>0.8	>0.8	>0.8	>0.9	>0.9	<0.08
Compliance	Yes	Yes	Yes	Yes	Yes	Yes	Yes

χ**^2^**, chi-square statistic; df, degrees of freedom; GFI, goodness of fit index; AGFI, adjusted goodness of fit index; NFI, normed fit index; TLI, Tucker–Lewis index; CFI, comparative fit index; RMSEA, root mean square error of approximation.

In general, the chi square to degrees of freedom ratio (χ^2^/df) should be between 1 and 3. If it is greater than 3, it indicates an under fitted model; if it is less than 1, it indicates an over fitted model. As shown in Table 5, in this study, χ^2^/df was 1.711, indicating a good model fit. The goodness of fit index (GFI) is a fitness index, and the adjusted goodness of fit index (AGFI) is an adjusted fitness index. When the GFI and AGFI values are close to 1, it indicates a better model fit; a typical threshold value is 0.8. The results in Table 5 show that in this study, GFI was 0.836 and AGFI was 0.803, indicating the good fit of the proposed model. The normed fit index (NFI) is the benchmark fit index and is equal to 1 minus the predefined model difference. The smaller the model difference, the closer the NFI value is to 1, and the better the model fit. Usually, the standard value for NFI is greater than 0.8. In this study, NFI was 0.871. The Tucker–Lewis index (TLI) is usually between 0 and 1. When TLI is 1, it means that the data fit the model perfectly. The general standard for TLI is 0.9. In this study, TLI was 0.934. The comparative fit index (CFI) is a comparative fit index and its value ranges from 0 to 1. When CFI is 1, it means that the data fit the model perfectly. The general standard for CFI is 0.9. In this study, CFI was 0.941, above the required standard. The root mean square error of approximation (RMSEA) refers to the mean square and square root of the asymptotic residuals. RMSEA is the ratio of the overall difference to the degrees of freedom and is typically less than 0.08. In this study, RMSEA was 0.058. Based on the above discussion, the EFA conducted in this study met the standards, indicating the overall fit of the proposed model.

To explore the factors that prompt tourists to choose a staycation, this manuscript designed and tested a staycation scale based on questionnaire data. Table 6 shows the CFA results. The three-dimensional measurement model fitted the data well. The factor loadings of most of the indicators in their respective measurement items were highly significant, and all of the Cronbach’s alpha coefficients were greater than 7, indicating the good reliability of the scale.

**TABLE 6:** CFA results.

Path			Estimate	*SE*	CR	*P*-value
RRP2	<—	RRP	0.715	0.081	11.200	0.000
RRP3	<—	RRP	0.764	0.085	12.161	0.000
RRP4	<—	RRP	0.737	0.088	11.632	0.000
RRP5	<—	RRP	0.737	0.086	11.626	0.000
RRP6	<—	RRP	0.754	0.083	11.972	0.000
RRP7	<—	RRP	0.740	0.086	11.682	0.000
BE1	<—	BE	0.868			
BE2	<—	BE	0.788	0.063	13.565	0.000
BE3	<—	BE	0.814	0.056	14.220	0.000
BE4	<—	BE	0.749	0.062	12.603	0.000
PB2	<—	PB	0.748	0.071	12.314	0.000
PB3	<—	PB	0.748	0.075	12.302	0.000
PB4	<—	PB	0.710	0.072	11.479	0.000
PB5	<—	PB	0.793	0.075	13.334	0.000
PB6	<—	PB	0.755	0.076	12.456	0.000
PB7	<—	PB	0.757	0.063	12.513	0.000
ATSC1	<—	ATSC	0.818			
ATSC2	<—	ATSC	0.777	0.078	12.397	0.000
ATSC3	<—	ATSC	0.822	0.075	13.344	0.000
ATSC4	<—	ATSC	0.791	0.073	12.703	0.000
BI1	<—	BI	0.875			
BI2	<—	BI	0.768	0.069	12.791	0.000
BI3	<—	BI	0.859	0.064	14.501	0.000
RRP1	<—	RRP	0.795			
RRP8	<—	RRP	0.821	0.083	13.380	0.000
PB1	<—	PB	0.815			
PB8	<—	PB	0.814	0.073	13.860	0.000

RRP, reduced risk perception; BE, benign envy; PB, perceived benefits; ATSC, attitude toward staycations; BI, behavioral attitude; SE, standard error; CR, composite reliability.

### Structural equation modeling

Structural equation modeling (SEM) is also a statistical method to analyze the relationship between variables based on the covariance matrix of variables, so it also becomes covariance structure analysis (Hair, 2009). SEM is a multivariate statistical analysis technique that organically combines multiple regression and factor analysis methods to automatically evaluate a series of interrelated causality (Yuksel et al., 2010). Structural equation modeling has similar uses with multivariate regression, but has more powerful functions, which is suitable for modeling under complex conditions such as hidden variables, independent variable correlation, existence variable error, multiple dependent variables and so on (Kyle et al., 2005).

**FIGURE 3:**
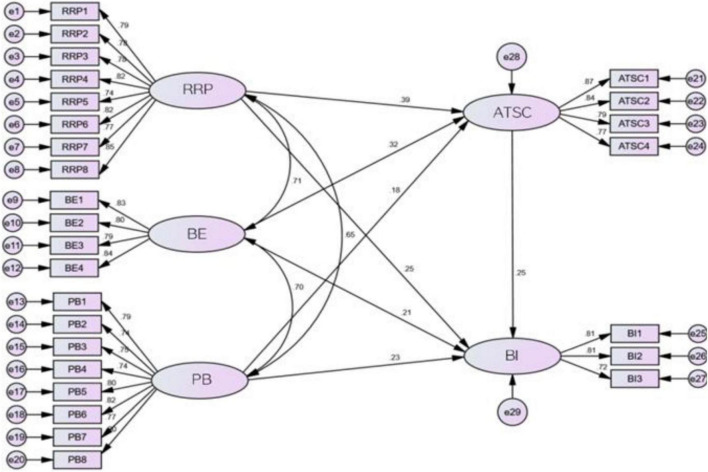
Structural model.

According to the theoretical model, the structural equation model is established in AMOS 21 by using reduced risk perception, benign envy, perceived benefit and attitude as independent variables and using behavioral intention as dependent variables (see Figure 3).

There are five latent variables in reduced risk perception, benign envy, perceived benefit, attitude and behavioral intention. Reduced risk perception has eight observation variables from RRP1 to RRP8) and error variance of eight observation variables. Benign envy has four observation variables from BE1 to BE4, and four observation variables error variance from e9 to e12, perceived benefits have eight observation variables from PB1 to PB2, and eight observation variables error variance from e13 to e20, attitude has four observation variables, and four observation variables error variance from e21 to e24, Behavior intention has three observation variables from BI1 to BI3, and three observation variables error variance. The estimated parameters have seven normalized path coefficient values, the normalized factor loading values of 27 observed variables, and 29 observed error variances.

When judging whether the structural equation model is valid or not, it is mainly measured by the calculation of some fitting indexes, in which χ^2^/df general requirements are less than 3. GFI fitness index, AGFI adjusted fitness index, NFI gauge fitness index, IFI value-added fitness index, CFI comparison fitness index are generally required to be greater than 0.9, indicating that the model adaptation ability is good, but the number greater than 0.8 indicates that the model is acceptable. RMSEA should be less than 0.08 to indicate better fitness. The model fits well. According to the following table, the χ^2^/df is 1.518, less than 3, GFI is 0.862, greater than 0.8, AGFI is 0.833, greater than 0.8, NFI is 0.895, close to 0.9, CFI is 0.961, greater than 0.9, RMSEA equals to 0.048, which is less than 0.08. It shows that the model fits well, model is therefore acceptable.

The standardized path coefficient of RRP to ATSC is 0.392 (*t*-value = 4.689, *p* = 0.000 < 0.01), which indicates that RRP has a significant positive effect on ATSC, that is, the higher the RRP, the higher the ATSC, so the hypothesis one is supported. The standardized path coefficient of BE to ATSC is 0.317 (*t*-value = 3.490, *p* = 0.000 < 0.01), which indicates that BE has a significant positive effect on ATSC, the hypothesis two is therefore supported. The standardized path coefficient of PB to ATSC is 0.180 (*t*-value = 2.253, *p* = 0.024 < 0.05), which indicates that PB has a significant positive effect on attitude, that is, the higher the PB, the higher the ATSC. The standardized path coefficient from ATSC to BI is 0.246 (*t* value = 2.523, *p* = 0.012 < 0.05), which indicates that ATSC has a significant positive effect on BI, that is, the higher the ATSC, the higher the BI, so the hypothesis four is supported; The standardized path coefficient of RRP to BI is 0.252 (*t*-value = 2.725, *p* = 0.006 < 0.01), which indicates that RRP has a significant positive effect on BI, that is, the higher the RRP, the higher the BI, so the hypothesis five is valid. The standardized path coefficient of BE to BI is 0.213 (*t*-value = 2.153, *p* = 0.031 < 0.05), which indicates that BE has significant positive effect on BI, that is, the higher the BE, the higher the BI, so the hypothesis six is supported. The standardized path coefficient of PB to BI is 0.232 (*t*-value = 2.763, *p* = 0.006 < 0.01), which indicates that PB has a significant positive effect on BI, that is, the higher the PB, the higher the BI, so the hypothesis seven is supported (see Table 7).

**TABLE 7:** Path coefficients between variables.

Path			Standardized path coefficients	*T*-value	*P*-value	Support
H1 ATSC	<—	RRP	0.392	4.689	***	Yes
H2 ATSC	<—	BE	0.317	3.490	***	Yes
H3 ATSC	<—	PB	0.180	2.253	0.024*	Yes
H4 BI	<—	ATSC	0.246	2.523	0.012*	Yes
H5 BI	<—	RRP	0.252	2.725	0.006**	Yes
H6 BI	<—	BE	0.213	2.153	0.031*	Yes
H7 BI	<—	PB	0.232	2.763	0.006**	Yes

*p < 0.05, **p < 0.01, ***p < 0.001.

## Discussion

For the eight RRP items, RRP2 to RRP8 had high explanatory power for higher-level items, with RRP8 having the greatest relevance to RRP. The reason that RRP1 was not the strongest correlation with RRP is probably that most of the respondents were aware of the severity of the spread of COVID-19, preventing them from wishing to travel. The other factors were likely to be driven by the belief that there is a lower likelihood of getting infected during a staycation and that a staycation is safer when adequate precautions are taken, therefore, the respondents were willing to go on staycations. The results are unfailing with those of a previous study (Lepp et al., 2011), indicating that positive feelings and travel intention can be generated when people’s risk perception is reduced.

For BE, being motivated to work hard was not a key factor, whereas the other three factors (“I compliment my friend”; “I wish it were me”; and “I want to follow my friend’s travel posts”) were important factors. The result of work enthusiasm may be related to the respondents’ income level, they may have reached a certain level of income and the cost of a staycation is not really high, so their decision to travel may not have been entirely dependent on their income growth. The other factors could have been influenced by the respondents’ need for social contact. In today’s society with highly developed social media, people want to emulate the extravagant lifestyle displayed on social media, so that their behavior comes across as a herd effect. These findings are consistent with social comparison theory and echo that of a previous study (Khan et al., 2017), in which BE was identified as a type of feeling in which a person is seen by others as having good results, accomplishments, or possessions. This feeling is expected to have a significant impact on people’s travel decisions.

For PB, PB2, to PB8 had high explanatory power for higher-level items, with PB8 having the greatest relevance to RRP. This result indicates that tourists make decisions based on the principle that the benefits should outweigh the costs, and they will only choose a staycation when they believe that the benefits are high enough. This demonstrates that “the economic man assumption” also applies to travel decisions.

For ATSC, three of the four items, namely funny, extremely pleasant, and positive, were identified as key factors, but enjoyable was not. For BI, excitement and family togetherness were identified as key factors, but escape was not. These results indicate that the purpose of a staycation is not to enjoy life and escape the present; the main goal is the pursuit of a fun, new, and sweet lifestyle. These results demonstrate that a staycation is an effective way to travel, similar to other types of vacation, which can reduce job stress and job burnout (Westman and Eden, 1997; Westman and Etzion, 2001, 2002; Westman et al., 2008).

## Implications

Given the current worldwide pandemic, destination management organizations should focus on identifying new marketing strategies to develop and increase local hospitality. This study sought to identify the antecedents of ASTC and subsequent BI. As an empirical study, the data were collected online via Wenjuanxing. The results of this study revealed that RRP, BE, and PB are the three main factors that influence people’s attitudes toward travel and their travel intention.

The results of this study have several theoretical implications. First, this study is the first to investigate staycations as a travel mode, and obtained unique data have obtained to examine the factors that drive tourists’ choice of staycations. Second, in this study, a theoretical framework, which lays the foundation for further exploration of staycations in future research is developed. Third, in the context of the COVID-19 pandemic, this study investigated the factors that stimulate tourism consumption in the tourism industry and combined psychological, communication, and tourism management theories to build a relevant and valid scale. In addition, to better investigate the perceptions and behavioral intentions of tourist, theoretical elaborations on the mindsponge mechanism were incorporated by this study in constructs development and refinement. Thus, it is established by the results of this study that mindsponge mechanism offers a reliable as well as valid theoretical framework to contribute to the understanding of the mechanism of a person’s attitude toward staycation. Although the mindsponge mechanism has been addressed in the literature for a while, its applications in the tourism area still emerging.

This study also has strong practical significance. First, hospitality authorities must take into account the factors that influence tourists’ intention to go on a staycation. The results of this study showed that people who choose a staycation appreciate the social value of travel, the positive energy and freshness that travel brings, and whether the travel destination is suitable for spending quality time with family members. Therefore, tourist attractions should create scenic, cultural, artistic, and other tourism projects to give these tourists what they want. Second, the individual characteristics and travel patterns of visitors are significant determinants of what prompts them to stay overnight when traveling. That is, understanding visitors’ characteristics and travel patterns is essential for hospitality authorities to decide on their marketing strategies to develop the local economy. At the same time, Information accessibility to staycation should be enhanced. New technologies such as the Internet and social media should be used to enhance local visibility and attract tourists through the advertising effect of the Internet. These efforts may lead to local economic growth. In addition, during the COVID-19 pandemic, staycation promotion information from various hospitality organizations is flooded in the internet, considering the importance of trust and the adverse effects of flooding information on the internet, hospitality policymakers should monitor and manage online staycation-related information thus tourists can make favorable subjective cost-benefit judgment. Apart from that, information management can also avoid misinformation spreading and loss of public trust (Vuong et al., 2022).

## Limitations

Although this study has made some contributions to the literature, it is not without limitations. One of the biggest limitations is that it can’t represent the total population of homestead travel, because the data is only collected online. Another limitation is that there is a single definition of staycation used in this study to ensure that distance and time can be easily measured and recognized by respondents. Future research could sample other staycation travel segments such as families or couples with children and those originated from Generation X. More parameters and more diverse samples can be leveraged for comparative study. Groups from different regions lead to group differences. In addition, holiday travelers can be interviewed for future research, asking them for their perception of these tour packages, and determining whether they can meet the needs of these travelers.

## Data availability statement

The raw data supporting the conclusions of this article will be made available by the authors, without undue reservation.

## Ethics statement

Ethical review and approval was not required for the study on human participants in accordance with the local legislation and institutional requirements. Written informed consent from the patients/participants or patients/participants legal guardian/next of kin was not required to participate in this study in accordance with the national legislation and the institutional requirements.

## Author contributions

All authors listed have made a substantial, direct, and intellectual contribution to the work, and approved it for publication.

## Footnotes

1
https://www.wjx.cn/
